# Hepatitis C virus, human immunodeficiency virus and pseudomonas phage PS5 triad share epitopes of immunogenic determinants

**DOI:** 10.1186/1743-422X-7-346

**Published:** 2010-11-26

**Authors:** Zhabiz Golkar, Nusrat Jamil

**Affiliations:** 1 No.102, Department of Microbiology, University of Karachi, Karachi, Pakistan

## Abstract

A lytic phage for *Pseudomonas aeruginosa *belongs to the *Myoviridea *family was isolated from urine for use in therapeutics. Pair of hepatitis C virus (HCV) primers highlighted segments on the genome of this phage. The sequence of these PCR products as well as the possible serological cross reactivity/relationship between HCV and the phage were investigated. One hundred HCV positive human sera were analyzed by ELISA. Ninety six well plates were coated with multiple epitopes of HCV proteins (Kit), phage and *Pseudomonas cells*. Initially the positive and negative control sera supplied in the test kit were used to evaluate the cross reactivity between the phage and anti-HCV antibodies. The results suggested a value over than 0.105 for a HCV positive reaction. Of the 100 HCV positive sera tested, sixty five and thirty percent showed cross reaction with phage lysate and *Pseudomonas aeruginosa, respectively*. High HCV antibody titer correlated to high cut off value for phage cross reaction, whereas no such correlation existed between HCV antibody titer and *Pseudomonas *cross reaction. The PCR products were sequenced and aligned with the HCV genome of H77. Sequence homology was detected in the 5', 3' UTRs and NS3 regions. Further these products showed similarity with HIV-1 Env, Pol & 3'LTR regions as well.

## Introduction

Hepatitis C virus (HCV) currently infects an estimated 3% of people worldwide encodes several proteins. HCV displays numerous interactions with the immune systems. Consequently a number of auto-antibodies are observed during the course of hepatitis C [1]. Many studies have detected the presence of antibodies reactive to a cloned host derived auto-antigen GOR and are highly correlated with the presence of antibodies to HCV. In chronic hepatitis C presence of these cross reactive antibodies is not merely due to sequence homology but also due to cross reactivity at the molecular level [2-5]. Antibody antigen reactions usually occur when an antigen combines with a corresponding antibody to produce an immune complex [6]. Specificity of this reaction refers to the ability of an individual antibody combining site to react with only one antigenic determinant or the ability of a population of antibody molecules to react with only one antigen. In general, there is a high degree of specificity in the antigen-antibody reactions [7]. However, cross reactivity refers to the ability of an individual antibody combining site to react with more than one antigenic determinant or the ability of a population of antibody molecules to react with more than one antigen [8]. Antigen antibody reaction is highly specific in some cases whereas cross reactivity is exhibited due to sharing of antigenic determinants by two unrelated microbes. For example, cross reactive anti HCV antibodies triggered by an epitope on HCV core protein which exhibits homology with auto antigen GOR 47-1 epitope. Similarly anti GOR antibodies, distinct from anti HCV core antibodies were revealed to have dual specificities. They target both the core gene product and host liver cell components [9]. Another cross reactive epitope shared by HCV NS3 protein and Influenza A (IV) virus. NS3- 1073 and influenza neuraminidase peptides displayed a high degree of sequence homology. These determinants are recognized by cytotoxic T lymphocytes with similar affinity. These heterologous antigens induce cross reactive CD8^+ ^T cells [10].

The reasons for the cross-reactivity between *Pseudomonas *phage lysate and human HCV positive sera are not known. We are the first to observe cross reactivity between HCV positive sera and newly isolated *Pseudomonas *phage antigens. The present study was undertaken to determine the reason for the cross reactivity or the non-specific reaction between HCV positive sera and the phage antigen. The findings are presented in this report.

## Materials and methods

### i. Determination of phage activity in clinical sample on bacterial lawn

Urine sample (50.ml) of a patient was centrifuged at 6000 rpm to remove solid matter and was then filter sterilized through a 0.45 μm membrane. 50 μl of the urine filtrate and 100 μl of 4 hrs young culture of *Pseudomona aeruginosa *were added to 3 ml of melted L.B soft agar and plaque assayed. All phages were purified by successive single plaque isolation until homogeneous plaques were obtained.

### ii. Isolation of phage from clinical specimen

One ml of the 4 hrs young culture of the respective hosts (*Pseudomonas aeruginosa (P5& P6 strains), E. coli, E. coli4MD, E. coli-N all local strains*) was mixed with 100 μl of urine, incubated for 2 hrs. at 37°C and centrifuged at 4000 rpm for 5 min. The pellet was washed twice with 500 μl of LB broth and suspended in 500 μl broth. *E.coli *lysogen cells exposed to UV for 1 min and incubated at 37°C for 2 hrs. The lysate was filtered, sterilized and plaque assay was performed.

### ii. Transmission Electron Microscopy

Particle morphology was studied by precipitating the lysate with PEG 6000 (Promega Co.) and NaCl to final concentration of 8% and 4%, respectively and incubated at 4°C overnight. The pellet resuspended in 100 μl of double de-ionized distill water. Four hundred (400) mesh carbon coated grids were negatively stained with 2% uranyl acetate for 30 seconds and examined in a GOEL-JEM-1200 EX II transmission electron microscope.

### iii. Desalting of lysate by micro dialysis

. Desalting of the phage suspension was carried out in an Eppendrof tube with the cap replaced with 2 cm of dialysis membrane that was held by a rubber band and floated in cold sterile distilled water in upside-down position. Water was changed after one hour and dialysis continued overnight at 4°C.

### iv. Coating of ELISA 96 wells plate by lysat

Phage PS5Φ concentrated by 0.95% PEG 8000 and 8% NaCl precipitation and suspended in 300 μl of sterile distilled water. The Phage suspension was then dialyzed, diluted in coating buffer (1:5) and coated on 96 wells plate as prescribed [11]. HCV positive serum (100 μl) was added to the coated wells and incubated for 1 hr. at 37°C. Assay was done as suggested by AutoBio Diagnostic Co.

### v. Coating of ELISA 96 wells plate by *Pseudomonas cells:*

The bacteria were scooped off from the lawn on BHI agar, washed several times with PBS buffer and a100 μl of cell suspension (1:10 in coating buffer) was added into each well of the ELISA plate and assay was performed.

### vi. Phage DNA purification

DNA was extracted by using Promega kit.

### vii. Polymerase Chain Reaction (PCR) amplification

Phage DNA was isolated from lysates PS5 Φ, and used as PCR templates. PCR products were generated using Taq DNA polymerase (Promega Co.) by following the manufacture's protocols. A range of primer sets were used (Table 1) The PCR conditions used were denaturation at 94°C for 4 min, followed by 35 cycles of denaturation at 94°C for 30 seconds, annealing temperature as 58°C (for C1& C3) and 60°C (for Ac2 & Sc2) for 30 seconds and finally extension at 72°C for 2 min. Amplified products were separated by 2% agarose gel electrophoresis and photographed.

**Table 1:** Primers used for Polymerase Chain Reaction (PCR)

**NO**.	primers	sequence	
**1**	C1	5'-AGGCGACACTCCACCATGGA-3'	58°C

**2**	C3	5'-TCACTCCCCTGTGAGGAACT-3'	58°C

**3**	Ac2	5'-GAGACGGGTATAGTACCCCATGAGAGTCGGC-3'	64°C

**4**	Sc2	5'-GGGAGGTCTCGTAGACCGTGGACCATG-3'	65.6°C

**5**	S7	5'-AGACCGTGCACCATGAGCAC-3'	60.3°C

**6**	A5	5'TACGCCGGGGGTCATGT-3'	72.3°C

**7**	Forward	5'-CCCGGGATCCGA-3'	58°C

**8**	Reverse	5'-ATGCCATCCCGGG-3'	58°C

### viii. DNA sequencing

Amplified PCR products separated by 2% agarose gel electrophoresis and fragments were selected and purified for sequencing. The selected PCR mixtures were prepared in 50 μl for each reaction and amplified products sequenced using the original amplification primers.

## Results

**Figure 1:**
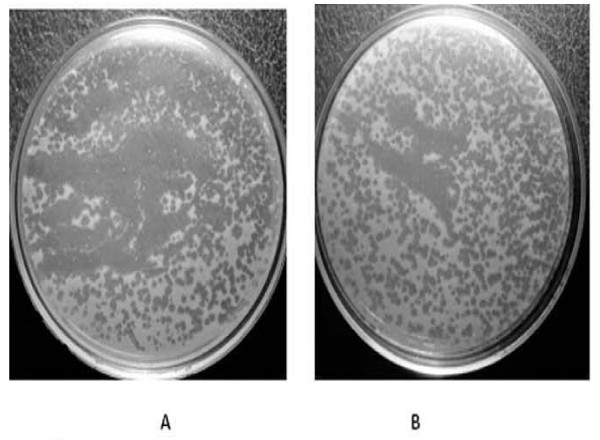
**Plaque Assay**: Plaque Assay of Lytic Phage on the Lawn of Different Strains of Multiple Drug Resistance *Pseudomonas spp*.

**Figure 2:**
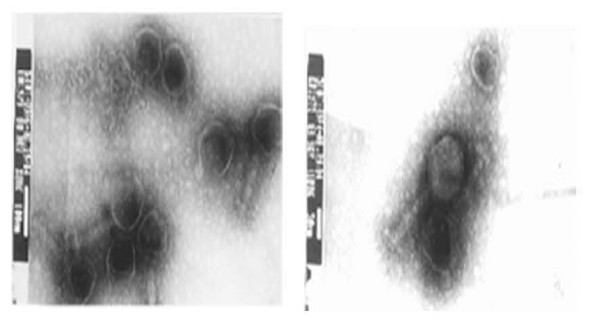
**Electron Micrograph**: Electron Micrograph of *Pseudomonas *Lysate PS5.

In this study, phages were isolated from the urine of a 24 years old female athlete with E. coli urinary tract infection (UTI). (Figure 1). Electron microscopy indicated the presence of *Siphoviridiae *and *Myoviridiae-*like morphology in the initial lysates prepared by using *Pseudomonas aeruginosa *strain (PS5) as host bacteria. Phage was further purified by single plaque assay (Figure 2). The presence of *E. coli *and lytic phages of Pseudomonas in the urine sample indicated the lysogenic status of *E. coli *which seems to be infected with a temperate phage which was lytic for *Pseudomonas *specie.

C1, C3 primers present in our collection were used contingently to highlight the PCR product from the amplified phage genome. Presence of discrete bands of 640-1169 bps (Figure four, lane 6 & 7; Table 2) warranted us to evaluate possible genetic or serological relation between phage and HCV. In order to confirm the possible serological cross-reactivity/relation, between phage and HCV +ve anti-sera, ELISA and PCR were performed.

**Table 2:** Quick Align Comparison of PCR Product of Phage Genome Homology with HCV and HIV Sequences in LosAlamos Database.

DNA	Primer	Size of PCR product	Sites on HIV-1 genome	Matched sequences size of PCR	Sites on HCV genome	Matched sequences size of PCR
PS5 Φ	Reverse	192	3'-LTR	10 nt	5'-UTR	150
	
	Forward	736	5'-LTR	7	5'-UTR	52
	
	Ac2	128	env	131	NS3	128
	
	Ac2	358	3'-LTR	30	3'-UTR	19
	
	Sc2	369	5'-LTR	19	3'-UTR	33
	
	Sc2	234	Pol	227	5'-UTR	2
	
	C1	1037	3'-LTR	12	3'-UTR	293
	
	C3	869	3'-LTR	92	5'-UTR	43

**Figure 3:**
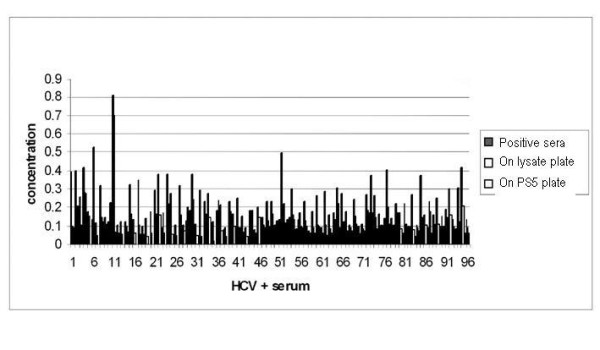
**Comparison of Cross reactivity of Phage PS5, *P5 *(whole cell) with Serum of HCV Positive Patients**.

Interestingly HCV positive control provided in the kit showed a strong reaction on phage coated plate (Figure 3). It was found that 83% of HCV positive sera tested reacted to the phage coated plate. This value is comparable to validity of HCV antigen coated plate. It has been shown that presence of antibodies in HCV positive sera is highly correlated with respective antigens Core/NS as indicated with ELISA (Figure 3)

Similarly the ranges for the *Pseudomonas aeruginosa *cross reactions with HCV positive sera (Figure 3) were found to be in the order of 30% sera. A plausible reason for the high *Pseudomonas *cutoff value for a HCV +ve serum (OD = 0.13) could be attributed to invasive *Pseudomonas aeruginosa *secondary infection in this patient. However a correlation between the HCV antibody titer and high cutoff value due to phage lysate was observed (Figure 3). This was further strengthened by the antigenic relation between phage and HCV in addition to the secondary infections of *Pseudomonas *in the HCV patients.

Results of ELISA suggest a serological relation between cascade (NS3, NS5, and Core) and *Ps*. phage (PS5 Φ). The enhanced ELISA cutoff value of phage lysate compared with *Pseudomonas aeruginosa *cell suggested significant cross-reactivity of HCV antibodies and phage antigens. High cut off value for cross reaction between phage antigen and 83% of the HCV sero-positive samples reflects the possible sequence similarities between antigen determinant region in the HCV genome and the phage genome.

**Figure 4:**
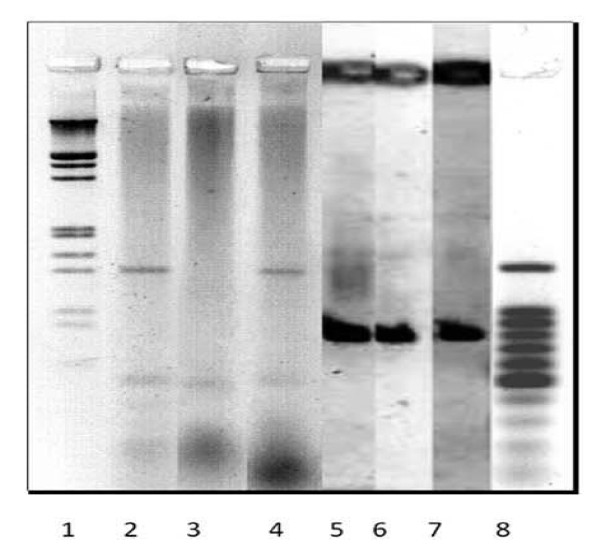
**Polymerase Chain Reaction (PCR)**.

**Figure 5:**
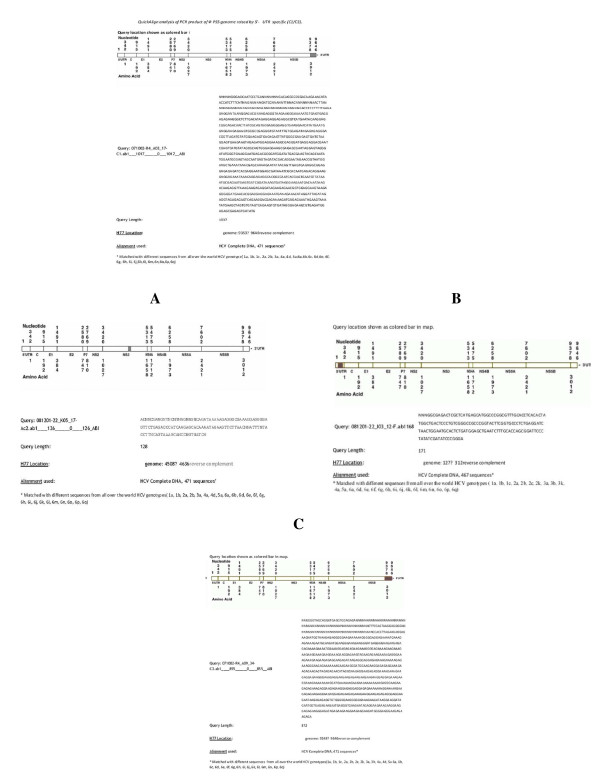
**QuickAlign**: QuickAlign Analysis of PCR Products of Φ PS5 Genome. Homologous with HCV Sequences in LosaAlamos-NCBI. Database. **A: QuickAlign**: QuickAlign analysis of PCR product of Φ PS5 genome raised by Universa (Ac2/Sc2) primers. **B: QuickAlign**: QuickAlign analysis of PCR product of ΦPS5 genome raised by Universal (Ac2/Sc2) primers. **C: QuickAlign**: QuickAlign analysis of PCR product of Φ PS5 genome raised designed (Forward) primer.(1^st ^band)

**Figure 6:**
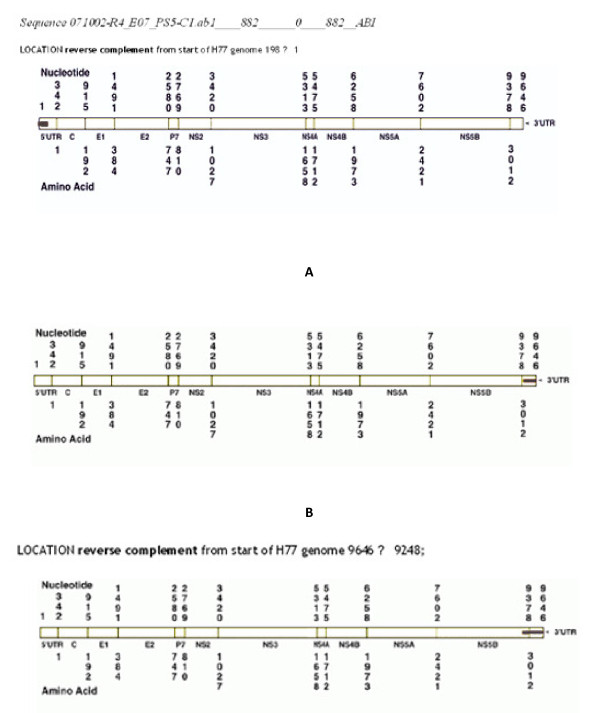
**Sequence Locator**: Sequence Locator Analysis of PCR Products of Φ PS5 Genome Homologous with HCV Sequences in NCBI Database A: *Sequence 071002-R4_E09_PS5. Sc2 ab1____746______0____746__ABI *B: *Sequence 071002-R4_AO9_PS5-C1.ab1____855______0____855__ABI*

The HCV 5'-UTR specific primers and universal primers (Ac2, Sc2, A5 and S7) were used to highlight the genome segment of *Pseudomonas *phage (PS5Φ) (Figure 4). These PCR products were sequenced and compared by Quick Alignment data exhibited the similarity among different HCV genotypes database (1a, 2a, 1b, 2b); (Figure 4). Gene locator software has highlighted reverse complement of nucleotide position (Similarity 90%) on 3'-UTR, 5'-UTR and NS3 region of H77 (shown as black bar in Figure 5A, C, 6A, B).

Preliminary data suggests that HCV 3'-UTR binds cellular proteins such as the 52 kDa La auto-antigen, the 57 kDa polypyrimidine tract-binding protein, and other proteins [12]. These results predict that isolated PS5 Φ may have regulatory machinery similar to HCV type translation-regulation. The similarity of phage translation-regulation regions and regulatory 5'and 3'-UTRs of HCV was substantiated by Quick Align and Gene locator analysis using non-HCV primers Reverse and Forward, (Table 2; Figure 5B) designed in our laboratory. This analysis showed variable matched size of PS5 Φ and 5'-UTR and 3'-UTR regulatory region of HCV genome.

It is worth noting that, 5'-UTR and the extreme end of the 3'-UTR of HCV have the lowest sequence diversity among various genotypes and subtypes. The relatively conserved nature of these regions is significant in their functional importance in the life cycle of the virus [13].

**Figure 7:**
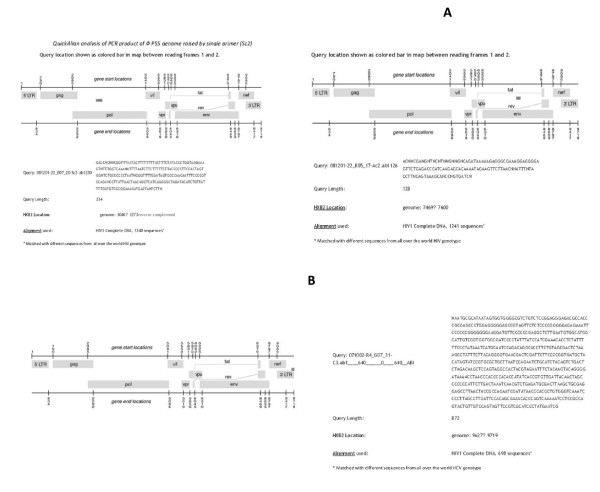
**QuickAlign**: QuickAlign Analysis of PCR products of Φ PS5 genome homologous to HIV sequences in LosAlamos database. *QuickAlign analysis of PCR product of Φ PS5 genome raised by single primer (Sc2) ***A: QuickAlign**: QuickAlign analysis of PCR product of ΦPS5 genome raised by pair primers (Ac2/Sc2) **B: QuickAlign**: QuickAlign: analysis of PCR product of ΦPS5 genome raised by single primer(C3)

Quick Align and Gene locator analysis of PCR products of Φ genome raised by HCV 5'-UTR specific primers have highlighted the region of phage that has similarity with domain I and II of 5'-UTR of HCV genome (Figure 5B &6). This un-translated, conserved region is involved in viral RNA translation and plays an important role in replication as well [14]. Analyses of the results have shown the similarity on region of 3'-UTR (Figure 5B, 6A, B), all these PCR products except (Figure 6A raised by Sc2) raised by C1 or C3 5'UTR specific primers. (Figure 5C &6B) showed homologous region extended into NS5B 3' end. These products were raised by C1+C3 pair. (Figure 5A &7A) indicated homologous region on NS3 of HCV and Env of HIV. These viral regions are highlighted by a PCR product of PS5Φ genome raised by Ac2 primer. (Figure 7) Exhibited relation between phage PCR product with HIV Pol gene. (Figure 7B) indicated the similarity between U5 regions of 3'LTR of HIV with PCR product raised by C3 primer of HCV. The comparative analysis confirmed that PS5 Φ has got the flexible regions on the genome, which is similar to the 5'-UTR IRES of HCV RNA, and may involved in the cap-independent translation.

## Discussion

Most of the PCR products raised by 5'UTR specific primers (C1, C3; Table 1, 2), highlighted from phage genome showed homology with 3' UTR region of HCV (Figure 5C, 6B). The entire 3'UTR is matched with these PCR products. However (Figure 5C &6B) exhibited homologous region extended into NS5B's 3' end. A stretch of 398 nts (reverse complement) of PS5Φ genome is similar to not only 3' UTR but 3' end of HCV genomic RNA. This homology has reflected significant value, since the conserved sequence and stem and loop structures of HCV function as cis acting signals that interact with viral and cellular proteins to initiate the synthesis of minus strand RNA during viral replication [15]. Recently Cristina [16], (2009) demonstrated the role of stem loop structure at 3' end of RNA, the 5BSL 3.2 motif which is embedded in cruciform structure at 3' end of NS5B coding sequence contribute to the 3 D folding of the entire 3' end of the HCV genome. It is essential in the initiation of replication. Presence of such functional/structural sequence in DNA genome of phage remains elusive and provides the incentive for investigation.

PCR product of PS5Φ genome raised by Ac2 HCV primer (Table 1 &2) exhibited sequence to NS3 at the HCV genome's location 4508←4636 (reverse complement) Figure 5B. Interestingly same PCR product is homologous to HIV genome at the location 7469→7600, which specify envelop gene (Figure 7). These 131 nts are highlighting the gp 120 outer-domain (OD) variable region comprised of several important immune epitopes. However V3 immuno dominance is the property of OD immunogens [17,18]. This stretch of Φ genome provides the compelling genetic evidence of sharing of some functional determinant between NS3 central domain and HIV gp120 region V3.

Large body of data indicated the implications of NS3/4A on adaptive immune response which cause inhibition of cytokines/chemokines [19]. Similarly gp120 interaction with cell chemokine receptor CCRS5 and CXCR4 are the key events for HIV-1 infection [20,21]. These results presented herein providing insight into possible mechanism by which HCV can acquires tissue tropism and can infect lymphoid cells i.e. monocytes/macrophages. Furthermore this shared region of HCV and HIV-1 is immunogenic can induce neutralizing antibodies [22,23]. These antibodies could be cross reactive too. Phillip [24] demonstrated the highly processive helicase activity on DNA by NS3. Presumably this may have the capacity to effect HIV DNA phase.

According to our results one of the PCR product raised by single primer Sc2 of HCV highlighting the PS5Φ genome segment homologous to HIV Pol gene at a location of HXB2 genome 3046←3273 (Figure 7). This segment of 227 nucleotides is a reverse complement and is complementary to the entire palm sub domain of HIV reverse transcriptase (RT). It is interesting to note that this sub domain of RT carries active site for replication [25-28]. Previous analysis of retroviral RT has indicated that palm domain in addition to finger and thumb sub domains facilitate the flow of genetic information moving in the reverse direction. It is worth to note that DNA genome of PS5Φ carries homologous sequence to palm sub domain of RT provide the insight into phylogenetic niches between this triad, HIV, HCV and DNA genome.

Interestingly PCR product raised by Ac2 primer highlighted two segments from PS5 Φ genome, as exhibited 2 bands on DNA gel (Figure 4, lane 2, 4); Analysis of the data from the first band showed similarity with HCV genome. A stretch of 128 nts exhibited similarity with NS3 region of HCV genome. The non-structural protein NS3, which is an essential component of HCV replication complex, is a protease that mediates NS2/3 cleavage. Serological cross-reaction between phage and HCV positive sera was found to be related to the presence of NS3 and this was further substantiated with HCV-Quick Align analysis of the PCR products (Figure 2). Clustal W alignment of the PCR products raised by Ac2 and retrieved NS3 sequence has confirmed that this homology is present close to N-terminal of NS3 region. Therefore, we predict that the genome of phage PS5 may code a protein having similar domain to NS3.

Epidemiological data shows that many HCV-infected patients are co-infected by HIV. Despite their remarkable differences, HIV and HCV share common characteristics (genetic, replicative, and pathological) that make real their interaction and facilitate the exacerbation of their related diseases [29]. It has been reported that hyper variable region of the NS1 protein of HCV may share similarities with the hyper-variable region of the envelope protein of human immunodeficiency virus HIV [30]. These results are in accordance to our finding as V3 region of envelop of HIV share determinant with NS3 of HCV. Serological tests and sequencing have shown that *Pseudomonas *phage (PS5 Φ) has similar antigenic epitopes to HCV and HIV variable immunogenic regions which contribute in complexity and diversity of these viruses. Previous reports have been demonstrated that a high proportion of blood donors exhibited false positive anti HCV antibodies [31]. Recently Jung-ah Kwon [32] used microarray assay for HCV detection and found same false results in few individuals. Therefore it can be concluded that PS5Φ presence in an individual may attribute to false positive results despite higher diagnostic accuracy of HCV/HIV infection. A healthy individual having this phage may unnecessarily receive interferon or antiviral drug. Therefore, diagnosis should be based strictly on HCV antigen detection and sequence analysis of the PCR products.

Based on computational analysis indicating 3'-UTR, 5'-UTR, NS3 of HCV and V3 of OD, palm sub domain of RT and U5 of 3' LTR of HIV are homologous with genome segments of PS5Φ. Which predicts that the genome of the *Pseudomonas aeruginosa *phage share similar regulatory stem and loop structure or common functional domains with the HIV/HCV genome. This can facilitate protein-protein interaction between prokaryotes and eukaryotes.

Horizontal transfer of genetic material between distantly related prokaryotes has been shown to play a major role in the evolution of bacterial genomes, but exchange of genes between prokaryotes and eukaryotes is not as well understood [33] and it cannot be ignored.

In this context, studies of retroviruses could be the best example illustrating the first demonstrated synthesis of DNA from RNA templates, a fundamental mode for transferring genetic material that occurs in both eukaryotes and prokaryotes. It has been noted that retrovirus infection which, introduces additional viral oncogenes into the cells, and transformed proteins leads to the conversion of normal cells to tumor cells [34,35].

Through this genetic mixing retrovirus and animal cells have been swapping genes for millions of years [36]. There is best circumstantial evidence suggesting retroviruses has been involved in other major evolutionary innovation too. McIntoch [37] (2008) proposed that the genes of DNA viruses were recruited in the evolution of eukaryotic machinery. Current work is consistent with this finding as PS5Φ has DNA genome carry a stretch of sequence homologous to entire palm sub domain of HIV RT, in fact exhibiting the evolutionary niches of most unrelated organism. Recent advances in understanding the molecular relation of host-pathogen and their interactions which highlighted the role in microbial evolution could play a significant role in the emergence of bacterial pathogens.

The exchange of genes between phage and virus should be visualized in this scenario. HIV/HCV patients are prone to any infections. *Pseudomonas *is a very common etiologic agent in infections particularly of the urinary and respiratory tracts. Therefore the body of HIV/HCV patient could be the re-union site for these prokaryotes i.e. phage and virus for the exchange of genetic material. Their flexible nucleic acids defiantly have their own modification to evade the nucleases of the host. HCV, HIV and PS5Φ triad proposed that fluxes of genetic material between prokaryotes and eukaryotes cannot be ruled out.

Finally, recent research by the virology and cell biology communities have developed the understanding of the co-infection (HCV/HIV) implication on complexity and diversity of HCV by ignoring other bacterial pathogens and their respective phages. Present study predicts that co-infection (HCV/HIV) with *Pseudomonas *phage PS5Φ may result in a higher rate of viral persistence. This may enhance the predisposing factors for complexity and diversity for HCV.

## Competing interests

The authors declare that they have no competing interests.

## Authors' contributions

ZG carried out complete bench work, participate in sequence analysis by applying bioinformatics tools and involved in partial drafting of manuscript. NJ conceived of the study, carried out design, inference of computational analysis and drafted the manuscript. Both authors read and approved the final manuscript.
